# Vascular graft infection by *Staphylococcus aureus*: efficacy of linezolid, teicoplanin and vancomycin systemic prophylaxis protocols in a rat model

**Published:** 2009-04

**Authors:** Erhan Atahan, Nurkay Katrancioglu, Ocal Berkan, Sinasi Manduz, Kasim Dogan, Yasemin Oztop, Ersin Tuncer, Hatice Ozer, Aynur Engin, Tulin Denizy Alta

**Affiliations:** Department of Cardiovascular Surgery, Cumhuriyet University School of Medicine, Sivas, Turkey; Department of Cardiovascular Surgery, Cumhuriyet University School of Medicine, Sivas, Turkey; Department of Cardiovascular Surgery, Cumhuriyet University School of Medicine, Sivas, Turkey; Department of Cardiovascular Surgery, Cumhuriyet University School of Medicine, Sivas, Turkey; Department of Cardiovascular Surgery, Cumhuriyet University School of Medicine, Sivas, Turkey; Department of Microbiology, Cumhuriyet University School of Medicine, Sivas, Turkey; Department of Pathology, Cumhuriyet University School of Medicine, Sivas, Turkey; Department of Pathology, Cumhuriyet University School of Medicine, Sivas, Turkey; Department of Clinical Microbiology and Infectious Diseases, Cumhuriyet University School of Medicine, Sivas, Turkey; Department of Pathology, Sivas State Hospital, Sivas, Turkey

## Abstract

**Objective:**

We investigated experimentally the *in vivo* prophylactic efficacies of linezolid, teicoplanin and vancomycin in subcutaneously implanted dacron graft infection caused by methicillin-resistant *Staphylococcus aureus* (MRSA).

**Materials and methods:**

Dacron grafts (1 cm^2^) were aseptically implanted into subcutaneous pockets that were surgically prepared in the backs of 50 rats. Ten of these rats were used as the control group (group I). Grafts in the remaining 40 rats were infected by inoculation of MRSA at the concentration of 2 × 10^7^ colony-forming units (CFU)/ml. Ten of these rats constituted the contaminated, untreated group II. The other three study groups comprising 10 rats each were contaminated and then treated with linezolid (group III), teicoplanin (group IV) and vancomycin (group V), respectively. All rats were sacrificed and the grafts were removed after seven days and evaluated.

**Results:**

The bacterial count decreased in the rats from the groups treated with linezolid, teicoplanin and vancomycin. The linezolid and teicoplanin groups, however, showed a significantly lower bacterial number than the vancomycin group (*p* = 0.009 and *p* = 0.01). The intensity of inflammation was highest in the contaminated, untreated group, as expected.

**Conclusions:**

Single-dose linezolid, teicoplanin and vancomycin for peri-operative prophylaxis may prevent bacterial growth in vascular graft infections. The effect of linezolid and teicoplanin seemed similar and their effect was greater than that of vancomycin.

## Summary

Prosthetic vascular graft infection is one of the most serious complications seen after vascular surgery. Prevention and treatment of prosthetic vascular graft infection has improved over time. However, mortality (up to 75% for intra-abdominal aortic grafts) and limb amputation (up to 70% for lower-extremities grafts) is still high.^1^-^3^ *Staphylococcus aureus* and coagulasenegative staphylococci are responsible for 70 to 90% of postoperative cardiac, thoracic and vascular infections.^4^ Prosthetic vascular graft contamination most frequently occurs during graft implantation or during the peri-operative period. *S aureus* and *S epidermidis* are two of the most common micro-organisms located on the skin.^5^ When vascular grafts contact the skin during the peri-operative period, these micro-organisms contaminate the vascular graft.

Asepsis and peri-operative administration of systemic antibiotics are essential to prevent graft infection. Many studies have shown that peri-operative systemic administration of antibiotics reduces the incidence of prosthetic vascular graft infections. However, gram-positive pathogens, particularly *S aureus*, are increasingly resistant to traditional antibiotics such as cefazolin.^6^ Linezolid, teicoplanin and vancomycin are logical options to prevent such infections. Linezolid is one of a new class of antibiotics called oxazolidinones that are chemically different from the currently available agents.^7^ Teicoplanin is a glycopeptide antibiotic and has an excellent bactericidal activity against penicillinase-producing and methicillin-resistant *S epidermidis* and *S aureus*.^8^ Vancomycin is used as a parenteral antibiotic therapy to treat staphylococcal infections.^9^

The aim of this study was to compare the *in vivo* efficacies of antibiotic prophylaxis with linezolid, teicoplanin and vancomycin in experimental graft infection caused by *Staphylococcus aureus* in a rat model.

## Materials and methods

The commercially available quality-controlled strain of methicillin-resistant *S aureus* ATCC 43300 (MRSA) used in this study was isolated from a clinical specimen submitted for routine bacteriological investigation in our microbiology laboratory.

Linezolid (Zyvoxid, Pfizer, Norway), teicoplanin (Targocid, Aventis, Turkey) and vancomycin (vancomycin hydrochloride, DBL, Mayne Pharma Plc, UK) were diluted in accordance with manufacturers’ recommendations, yielding 1 mg/ml stock solutions. Solutions of each drug were fresh on the day of assay.

The antimicrobial susceptibilities of methicillin-resistant *S aureus* (MRSA) strains were determined using the micro-broth dilution method, according to the procedures outlined by the National Committee for Clinical Laboratory Standards.^10^

## Rat model

This study was approved by the Animal Ethics Committee of our institution. Fifty adult female Wistar rats (weight range, 200–250 g) were used. All rats had free access to standard rat feed and tap water. The rats were randomised into five groups after subcutaneous graft implantation which is described below: group I, no graft contamination and no antibiotic prophylaxis; group II, MRSA contamination but no antibiotic prophylaxis; group III, MRSA contamination, with linezolid prophylaxis; group IV, MRSA contamination, with teicoplanin prophylaxis; and group V, MRSA contamination, with vancomycin prophylaxis. Linezolid, teicoplanin and vancomycin were administered as a single intraperitoneal dose of 10 mg/kg, as stated previously in the literature, 30 min before implantation of the graft.^11^,^12^

## Surgical technique

Intraperitoneal ketamine hydrochloride (90 mg/kg, Ketalar, Pfizer, Turkey) and xylazine hydrochloride (3 mg/kg, Rompun, Bayer, Turkey) were administered to attain sufficient anaesthesia before the experiment, and additional doses were applied when necessary. The rats’ backs were shaved and the skin was cleaned with 10% povidone iodine solution. One subcutaneous pocket was made on the right side of the median line of each rat by means of a 1.5-cm incision. Aseptically, 1-cm^2^ sterile, gelatin-sealed dacron grafts (Gelseal; Sulzer Vascutek Ltd, UK) were implanted into the pockets and 1 ml saline solution containing MRSA strain at a concentration of 2 × 10^7^ CFU/ml was inoculated onto the graft using a tuberculin syringe to create a subcutaneous fluid-filled pocket. The pockets were closed with 5/0 polypropylene sutures (Dogsan Ltd, Turkey). The animals were returned to individual cages and thoroughly examined daily. All grafts were explanted seven days after implantation.

## Assessment of infection

The explanted grafts were placed in sterile test tubes, washed in sterile saline solution, placed in test tubes containing 10 ml of phosphate-buffered saline solution and sonicated for 5 min to remove the adherent bacteria from the grafts. Quantification of the viable bacteria was performed by preparing serial 10-fold dilutions (0.1 ml) of the bacterial suspensions in 10 mM buffer to minimise the carryover effect and culturing each dilution on blood agar plates. All plates were incubated at 37° C for 48 h and evaluated for the presence of MRSA. The organisms were quantified by counting the number of colony-forming units per plate. The limit of detection for this method was approximately 50 CFU/cm^2^ of graft tissue.

## Histopathological examination

Graft material, which had been implanted into the subcutaneous tissue, was removed with its surrounding skin and subcutaneous tissue from each rat for the histopathological examination. These tissue samples were fixed in 10% formaldehyde solution for at least 24 hours and then embedded in paraffin blocks. Slide sections of 5-μm thickness were taken from paraffin blocks and stained with haematoxylen-eosin stain. The stained slides of skin and subcutaneous tissue were examined blindly without any knowledge of the group from which it came, for the parameters: intensity of inflammation, intensity of fibroblastic proliferation, neovascularisation, amount of oedema and amount of collagen, using the modified 0–4 Ehrlich and Hunt numerical scale.^13^ These parameters were evaluated separately using the histopathological grading scale shown in Table 1.

**Table 1:** Histopathological Grading Scale

0	No evidence
1+	Occasional evidence
2+	Light scattering
3+	Abundant evidence
4+	Confluent cells or fibers

## Statistical analysis

Data were presented as mean ± standard deviation (SD). The data obtained from quantitative culture and histopathological evaluations were analysed with the Kruskal-Wallis test, and multiple comparisons between the groups were performed with the Tukey test. A *p*-value of 0.05 or less was considered statistically significant.

## Results

None of the animals in any group died or had clinical evidence of drug-related adverse effects, such as local signs of perigraft inflammation, anorexia, diarrhoea or behavioural alterations. However, polydipsia was observed in the vancomycin group.

None of the animals included in the control group had either anatomical or microbiological evidence of graft infection. By contrast, all rats in the untreated, contaminated group demonstrated graft infection, evidenced by the quantitative culture results of 1.2 × 10^5^ ± 9.7 × 10^4^ CFU/cm^2^. The quantitative graft cultures of the linezolid, teicoplanin and vancomycin groups each demonstrated different counts of bacterial growth (Table 2). The results from the linezolid-, teicoplanin- and vancomycin-treated groups showed significantly lower bacterial numbers compared with the untreated, contaminated group (*p* < 0.001 for all). There was no statistically significant difference between the linezolid and teicoplanin groups (*p* = 0.12) but the linezolid- and teicoplanin-treated groups showed significantly lower bacterial numbers compared with the vancomycin group (*p* = 0.009 and *p* = 0.01).

**Table 2:** Quantitative Microbiological Results Of The Study Groups

*Groups*	*Intraperitoneal pre-operative drug*	*Quantitative graft culture (CFU/cm2)*
Group I	–	No growth
Group II	–	1.2 × 10^5^ ± 9.7 × 10^4^ *
Group III	Linezolid	1.1 × 10^1^ ± 0.9 × 10^1^
Group IV	Teicoplanin	2.5 × 10^1^ ± 0.7 × 10^1^
Group V	Vancomycin	1.8 × 10^2^ ± 3.2 × 10^1^ **

*p < 0.001 vs groups III, IV and V. **p < 0.01 vs groups III and IV.

The intensity of inflammation was highest in the untreated, contaminated group (Table 3). In the evaluation of the intensity of fibroblastic proliferation, the control group had significantly lower proliferation rates than the other groups (Table 3). Amount of collagen was significantly lower in the untreated, contaminated group compared with the other groups (Table 3). With regard to neovascularisation, all groups were comparable (Table 3). Amount of oedema was significantly higher in the linezolid, teicoplanin and vancomycin groups compared with the untreated, contaminated group (Table 3).

**Table 3:** Histopathological Findings Of The Study Groups

*Groups*	*Intensity of the inflammation*	*Intensity of fibroblastic proliferation*	*Neovascularisation*	*Amount of oedema*	*Amount of collagen*
Group I	1.70 ± 0.48	1.60 ± 0.51^b^	2.80 ± 0.42	2.60 ± 0.51	2.90 ± 0.31
Group II	2.80 ± 0.42^a^	2.40 ± 0.51	2.70 ± 0.48	0.70 ± 0.82^a^	2.00 ± 0.00^a^
Group III	1.54 ± 0.52	3.00 ± 0.00	2.90 ± 0.30	1.72 ± 0.90	2.81 ± 0.40
Group IV	1.36 ± 0.50	2.81 ± 0.40	2.81 ± 0.40	1.45 ± 0.68	2.72 ± 0.46
Group V	1.60 ± 0.69	3.0 ± 0.00	2.80 ± 0.42	1.70 ± 0.67	2.50 ± 0.52

^a^*p* < 0.05 vs groups I, III, IV and V. ^b^*p* < 0.05 vs groups II, III, IV and V.

## Discussion

Graft infections are a serious complication of vascular surgery. All prosthetic vascular grafts are to varying degrees susceptible to infection, either via direct contamination during implantation or bacteraemia after the operation. MRSA is increasingly becoming a problem in cardiovascular surgical units and most graft infections are believed to occur at the time of graft insertion.^1^,^14^ Prevention of prosthetic vascular graft infection is essential, as infection usually results in graft excision, with the resultant high morbidity and mortality rates.

Several studies have demonstrated that systemic antibiotic prophylaxis reduces the incidence of prosthetic vascular graft infection, but does not prevent it. Due to the emergence of resistance to antibiotics, a variety of antibiotics and prophylaxis protocols are under investigation. Linezolid, teicoplanin and vancomycin seem the obvious options to prevent such infection. We therefore tested this hypothesis using the rat as an animal model, which is the preferred method of testing antibiotic sensitivity prior to human testing.

The glycopeptides vancomycin and teicoplanin are bactericidal agents with the ability to inhibit bacterial cell wall synthesis. Teicoplanin is preferable to vancomycin, since it has fewer side effects, e.g. nephrotoxicity and ototoxicity, and therapeutic drug monitoring is usually unnecessary.^15^ In this study, vancomycin and teicoplanin decreased the bacterial count significantly.

Maki *et al*.^16^ suggested that vancomycin deserves consideration for inclusion in the prophylactic regimen for prosthetic valve replacement and vascular graft implantation, to reduce the risk of implant infection with methicillin-resistant coagulase-negative staphylococci and enterococci and for all cardiovascular operations in centres with a high prevalence of surgical infection with methicillin-resistant staphylococci or enterococci. However, in this study, we found that teicoplanin showed significantly better efficacy in lowering bacterial count compared to vancomycin.

Antrum *et al.*^17^ suggested that teicoplanin exhibits good penetration into ischaemic tissue, which is desirable for prophylaxis in vascular surgery. Similarly, Turgut *et al.*.^11^ concluded that teicoplanin was effective in the reduction of prosthetic vascular graft infections. Previously, we have shown that the efficacy of teicoplanin was greater than that of vancomycin and cefazoline.^12^

Recent studies reported by Hiramatsu *et al*.^18^ have shown *S aureus* isolates with reduced susceptibility to glycopeptides. Vancomycin and teicoplanin are glycopeptides but linezolid is in a new class of antibiotic called the oxazolidinones.^5^,^7^ Linezolid has a unique mechanism of action. It inhibits bacterial protein synthesis by binding to the 50S ribosomal subunit; this binding prevents the formation of a functional initiation complex in bacterial translation systems. Linezolid is active against a number of gram-positive bacteria. It has excellent activity against both methicillin-sensitive and MRSA.^19^,^20^ In an *in vitro* investigation, Edminston *et al.*^21^ suggested that linezolid and daptomycin appear to exhibit potent antimicrobial activity against deviceadherent strains of staphylococci. It has been reported that linezolid possesses an *in vitro* activity against MRSA, which is comparable to or even better than those of the glycopeptides vancomycin and teicoplanin.^22^ In this study, linezolid decreased the bacterial count more efficiently. Linezolid showed significantly lower bacterial counts compared to vancomycin, though, it was not more effective than teicoplanin.

Prevention or treatment of infection is important for successful wound healing. The presence of locally destructive bacterial colonisation is an important factor in delayed wound healing, even though it does not produce deep infection. Tissue repair is negatively influenced by cytolytic enzymes, free oxygen radicals and other pro-inflammatory mediators which are produced due to the continuous neutrophile flow seen in the inflammatory response Chronic wounds always contain bacteria and these micro-organisms attach to the tissue and form colonies by multiplying in the wound. However, wound healing is only slowed down if the number of bacteria and the bacterial virulence increase tremendously. Delayed wound healing appears to be due to bacterial overload, a critical mass of colonisation, local infections and covered infections.

In the present study, parameters of wound healing such as intensity of inflammation, intensity of fibroblastic proliferation, neovascularisation, amount of oedema, and amount of collagen were evaluated histopathologically. The intensity of inflammation was found to be highest in group II, in which *S aureus* was inoculated onto the graft area without any antibiotic administration to the rats. Group II also showed significant decrease in fibroblastic proliferation, oedema and collagen synthesis when compared with the other groups. These results showed that inflammation had a negative effect on wound healing, as has been reported in the literature.^23^-^25^ With regard to neovascularisation, there was no significant difference between the groups, showing that neovascularisation was not as influenced by the infection as was the intensity of inflammation. The groups with antibiotic treatment showed no significant difference between them for all the parameters of inflammation.

Two limitations of this study were: in our model, wound reaction to a foreign body (implanted graft) was monitored, rather than the healing process with vascular prostheses; and the relatively small sample size of our study did not allow us to draw firm conclusions. However, we propose, in the light of our findings, that the use of single-dose linezolid, teicoplanin and vancomycin for peri-operative prophylaxis may prevent MRSA growth, which is a feared complication following surgery. The effect of linezolid and teicoplanin was similar and more potent in subcutaneous tissues than that obtained with vancomycin.
